# Legal Needs Arising in Mental Health Settings and Staff Capability and Support to Respond

**DOI:** 10.5334/ijic.7693

**Published:** 2024-03-21

**Authors:** Suzie Forell, Sarah O’Connor

**Affiliations:** 1 Health Justice Australia, Australia; 2 University of NSW Faculty of Law and Justice, Australia; 3 University College London School of Laws, United Kingdom; 4 Neami National, Australia

**Keywords:** mental health, staff capability, legal need, social determinants, health justice partnership

## Abstract

**Introduction:**

Legal issues are known to affect and be affected by mental health. But to what extent do legal issues surface in mental health settings and what do staff feel they need to support clients experiencing these issues? These questions were explored by a national mental health service interested in the potential for health justice partnership with local community based legal services.

**Methods:**

A survey of 999 frontline staff of a national mental health organisation. 146 staff (15%) responded from 70 service sites across Australia, including peer support workers (47%), support workers (20%), team leaders (17%) and clinicians (15%).

**Results:**

Staff identified a wide range of legal issues experienced by their clients (commonly referred to by staff as consumers), most commonly credit, debt and social security issues, housing, family law and family violence. Two-thirds (67%) of respondents indicated that they spent around 50% *or more* of their time ‘responding to these types of issues’. Respondents indicated that they need more support to address legal issues facing their clients, particularly more knowledge of other services, connections with professionals in other organisations and connections with community. They also felt they could benefit from additional processes, tools, and resources, and time to manage their case load.

**Originality:**

While there is an emerging field of research exploring the legal capability of citizens, this study explores what mental health service staff feel they need to support consumers experiencing legal issues that can interact with mental health.

## Introduction

### The intersection between legal need and mental health

Since the World Health Organisation’s 2008 Commission on Social Determinants of Health, evidence has continued to grow about the influence of social and environmental factors in driving poor health outcomes for individuals and communities. A person’s health can be undermined by factors such as substandard housing, limited income, insecure employment, vulnerability to violence, social exclusion, and criminal justice. There is a social gradient to this impact, where overall, the lower a person’s socioeconomic position, the worse their health [1].

Over the same period, legal needs and access to justice research globally has identified the concentration of legal need among those experiencing disadvantage; the clustering of legal issues together, and with other life and health issues; barriers to help seeking, and inaction about legal issues [2,3,4,5] ‘Legal issues’ are problems that have a legal dimension that a lawyer can assist with. ‘Legal need’ arises when these issues are not addressed.

This evidence also indicates that people experiencing mental health issues are among those most vulnerable to having unmet legal need and who face significant personal and systemic barriers in accessing and using legal help. It also points to the bidirectional impact of mental health and legal problems: issues such as spiralling debt, insecure or inappropriate housing and family breakdown can all contribute to poor mental health, and poor mental health can in turn escalate these issues [6,7,8].

### The role of health professionals in addressing legal issues that affect health

Legal needs research has identified the key role of non-legal professionals, also described as ‘trusted intermediaries’ in linking people experiencing legal issues with the legal help they would otherwise not access in a timely manner or at all [3,9]. This is because people with legal issues are more likely to be in contact with trusted workers in health and community settings, than directly with legal professionals and services [2]. Contributing to this, many people do not necessarily identify legal issues relating to housing, credit and debt, family violence and the like – as issues that lawyer can help them with [2,3,8,9]. The skills, knowledge, mindset and resources to identify and appropriately respond to legal issues is described in the access to justice literature as ‘legal capability’ [10]. The phrase is most commonly used in reference to those experiencing legal need. However, to effectively link people experiencing legal needs to the professionals that can assist them, intermediaries also require ‘legal capability’.

A Canadian study on the role of trusted intermediaries identified that, to effectively identify legal need and act as a pathway to legal assistance, community workers required a range of support: training, tools, resources and connections and partnerships [9 p. 6].

Clarke (2017) found that mental health professionals provided clients with support including identifying financial difficulties, helping clients to access debt advice services and address their financial issue, as well as providing specialist support to address the underlying psychological causes and consequences of financial difficulties [11 p. 5]. The professionals surveyed and interviewed included social workers, mental health nurses, support workers, occupational therapists, psychotherapists, psychologists, and psychiatrists working in community mental health teams, crisis teams and acute hospitals. The study identified the challenges faced by practitioners in undertaking this role, including lack of knowledge of, access to, and connection with, specialised financial advice services; lack of clarity around the limits of their own roles in helping their clients with these issues; or feeling the need to step beyond their expertise and role to address a critical need [11 p. 30]. The study recommended training around financial difficulty and its link to mental health, more streamlined ‘warm referral’ channels to appropriate expertise, and a specialised, co-located service for those with the most severe needs [11 pp. 40 & 38].

Health justice partnership (HJP) is a service response to intersecting need, whereby legal help is integrated into health care settings and teams to address socio-legal issues that contribute to poor health. While they vary by context, HJPs commonly provide on-site legal help to individual clients, and enable health practitioners to directly consult with legal professionals about problems clients are experiencing (secondary consultation). They can offer cross-disciplinary training for partnering practitioners, facilitate ‘warm’ referrals, and provide the opportunity for partners to work collaboratively to support shared clients. Recognising the important role of health staff as intermediaries, partnerships build the capability of health staff to identify and address legal issues that are already arising in their practice [12].

## Methodology

This study was part of a project to develop HJPs in three Neami National sites. Neami National is a community-based organisation providing local services around Australia in mental health, homelessness and suicide prevention support. Prior to establishing the HJPs, a survey of frontline survey staff was conducted to provide a baseline insight into the types of legal issues facing clients using these services, and the current ‘legal capability’ of staff to address those needs. As most of these services describe clients as ‘consumers’, we used this term in the survey and will use it to describe the results. We acknowledge the range of terms to describe this population, including consumer, service user, and guest. We note that language is contested, and all terms have limitations. We use the term ‘clients’ when referring to the broader literature and context.

The study was conducted by Neami National and approved by the Neami National Research and Evaluation Committee and the University of New South Wales (UNSW) Human Research Ethics Committee (HREC).

Frontline service site staff (n = 999) were invited to participate in the online survey. This included peer workers, clinical staff, operational support staff, site managers and team leaders, and a range of other support workers including wellbeing coaches, Aboriginal health officers, registered nurses, employment specialists and support coordinators. Staff were informed of the study through internal communications such as webinars, newsletter updates and emails. Participants were provided with electronic ‘Participant Information and Consent Forms’ via email along with the survey link. The voluntary nature of participation was made explicit in all communications.

Survey questions focussed on:

- Types of legal issues that staff see in their work
- Time spent addressing legal issues
- Whether staff felt they had the resources to address legal issues in their work
- History of consulting with lawyers
- Current referral relationships with other legal services.

Descriptive statistics for each survey question were generated using Microsoft Excel. Additionally, we undertook quantitative data analysis using ‘R’ statistical analysis software to explore whether a range of factors influenced participant’s responses to survey questions. Survey responses were analysed by role type, service type and history of consulting with a lawyer.

## Results

146 staff completed the survey which corresponded to a national response rate of 15%. Response rates for each state and territory were: Northern Territory (30%), Western Australia (16%), NSW (15%), Victoria (14%), Queensland (10%) and South Australia (10%).

The survey respondents worked across 70 service sites in every Australian state and territory that the organisation has services. In terms of role type, 47% of all respondents were peer support workers, 20% were support workers, 17% managers/team leaders and 15% clinical workers. Respondents (n = 144) reported their main worksite (service type) as:

- Community-based mental health support. These services include mental health outreach, intake and assessment and/or service navigation, consortium management/subcontracting, group programs, residential programs (47%).
- Recovery-oriented clinical mental health services. These services include short-term subacute residential services, subacute community outreach, recovery focussed clinical services, and Adult Mental Health Centres (42%).
- Housing and homelessness services. These services include street outreach, wrap around support, transitional residential and support, and low intensity housing support (12%).

### Previous experience with lawyers

**Figure 1:**
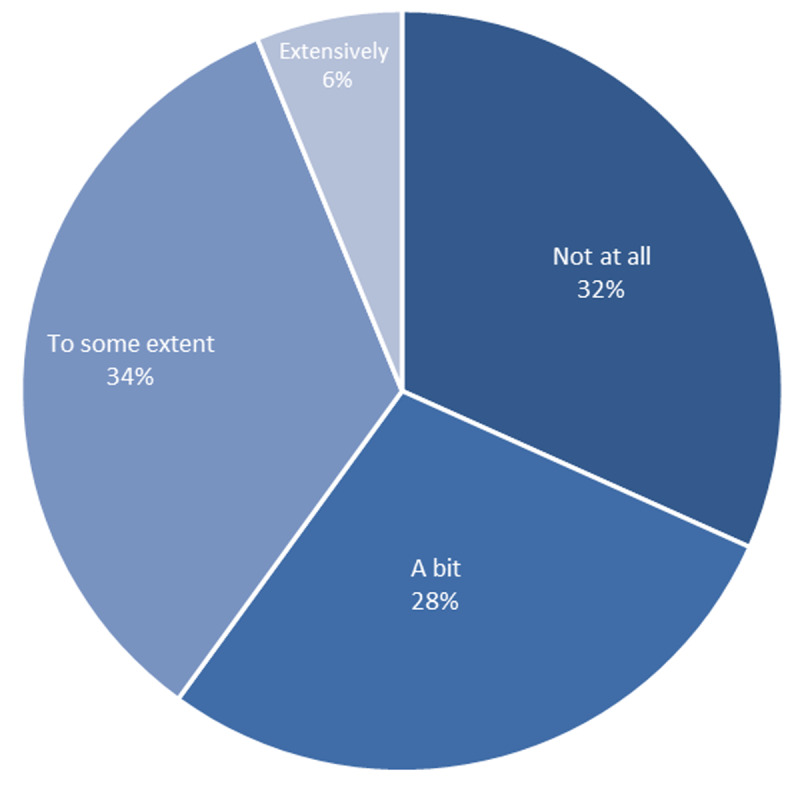
Proportion of respondents who had consulted with lawyers to support consumers.

To understand worker’s current connections to legal services we asked ‘In your current or previous roles, have you ever consulted with lawyers to support consumers?’ Figure 1 indicates that one-third (32%) of respondents had never consulted lawyers to support their consumers while a further 28% said they had done so ‘a bit’. Another third had done so to ‘some extent’ (34%). Six per cent had done so ‘extensively’.

### Differences between service types

Differences between service types were tested. Using Analysis of Variance (ANOVA), results showed significant differences between housing and homelessness services and the other two service types. Respondents from housing and homelessness services reported significantly more frequent consultation with lawyers than those from community-based mental health support services and recovery-oriented clinical mental health services. This finding was limited by the small sample size and large variability of scores for housing and homelessness services.

Respondents were also asked if they already had referral relationships with legal services. More than one quarter (27%) said they had no referral relationships with legal services, and 13% did not know. Nearly half (49%) said they had informal referral relationships only, while 11% said they had formal referral relationships (only 6%) or formal and informal relationships (5%).

No statistically significant differences were found by role type, state or time working in the organisation based on previous experiences working with lawyers.

### Legal need in mental health care settings

With people experiencing mental health issues more vulnerable to legal issues, we explored whether the legal issues of consumers are visible to mental health practitioners. Respondents were asked how often the following list of issues arise in their work with consumers.

- Money issues (e.g. debts, fines, payday loans and mortgage stress)
- Social security/Centrelink issues (e.g. breaches and eligibility)
- Housing/tenancy issues (e.g. eviction and disputes with landlords)
- Family/relationship issues (e.g. separation and child access)
- Domestic or family violence (e.g. risk to self or family)
- National Disability Insurance Scheme/National Disability Insurance Agency (NDIS/NDIA) issues (e.g. access to scheme and appeal process)
- Victim of crime issues (e.g. fraud and abuse/neglect)
- Care and child protection issues (e.g. risk of child removal and protection orders)
- Crime issues (e.g. court appearances as a defendant and traffic infringements)
- Discrimination, harassment, or bullying issues (e.g. in school, housing, and employment)
- Issues relating to mental health legislation
- Employment issues (e.g. unfair dismissal and poor work conditions)
- Other issues (e.g. consumer issues, scams, and car accidents)
- Visa and immigration issues (e.g. family and refugee visas).

**Figure 2:**
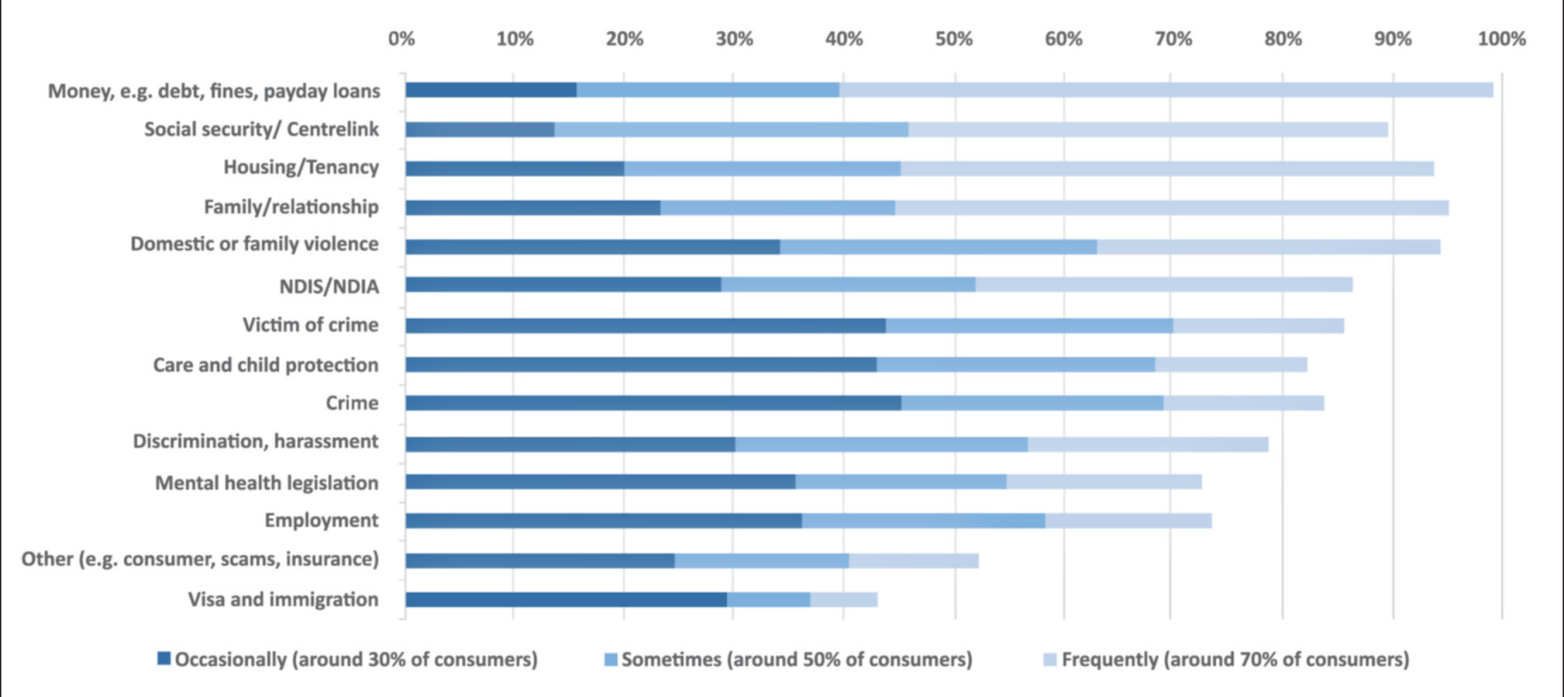
**Legal issues identified by mental health staff** *(Legal issues ordered by ‘Sometimes’ and ‘Frequently’ combined (50% or more))*.

Response options were ‘not at all’, ‘occasionally (about 30% of consumers)’, ‘sometimes (around 50% of consumers)’ or ‘frequently (around 70% of consumers)’ (Figure 2).

Virtually all respondents (99%) said that legal issues relating to money came up *at least* occasionally with the consumers they support, with 60% indicating that this was ‘frequently’ the case. Other legal issues seen at least occasionally by more than 90% of respondents were: family/relationship issues (95%); family violence (95%); Housing/tenancy (94%) and social security issues (90%).

In addition to money issues, other ‘frequently’ raised issues were family/relationship issues (51%), housing or tenancy issues (49%) and social security issues (44%), with 32% indicating that family violence issues arose frequently. Issues relating to the National Disability Insurance Scheme were reported to be raised frequently by more than one-third of respondents.

Victim of crime issues were seen by 86% of respondents, while criminal law (Crime) issues were seen by 84% of respondents. Crime was most commonly seen ‘occasionally’ (45%). Differences between service types were tested. Differences between service types were evaluated using a Kruskal-Wallis test. Results showed significant differences between housing and homelessness service and other service types. Criminal law issues were identified significantly more frequently by respondents who worked in housing and homelessness services than those from community-based mental health support services and recovery-oriented clinical mental health services.

No statistically significant differences were found in other survey responses when analysed by role type or service type, which may be due to the small number of respondents.

Overall, 70% of staff said that they saw 12 of these 14 issue types at least occasionally in their practice. This is consistent with existing legal needs research which identifies the vulnerability of people with mental health issues to multiple legal issues, particularly when they also experience additional indices of disadvantage [13].

### The time workers spend responding to these issues

Two-thirds (67%) of respondents indicated that they spent around 50% or more of their time ‘responding to these types of issues’. Within this, 36% indicated that this took around 70% of their time.

### Do workers have what they need to address legal issues?

With consumers presenting to services with a range of socio-legal issues in their lives, we asked workers whether they ‘had enough’ of what they needed to support consumers with these issues, or whether they needed ‘a bit more’, ‘some more’ or ‘a lot more’. We explored aspects of their ‘legal capability’ (skills, knowledge and mindset to see and address legal issues) [14] as well as features of their work environment (time, remit, processes, relationships).

Respondents most commonly said they needed more ‘connections with professionals in other organisations’ (88%, with 51% saying they need some or a lot more). Most said they also needed more ‘connections with local communities’ (86% with 50% saying they needed some or a lot more).

In terms of personal capability, respondents indicated that they needed at least a bit more:

- knowledge of other services that can assist consumers with these issues (84%, with 44% saying they needed some or a lot more)
- trust in other services (76%, 38% some or a lot more)
- knowledge about the types of legal issues that consumers may face (73%, 40% some or a lot more)
- appropriate skills and experience (68%, 33% some or a lot more)
- confidence to link consumers to help beyond my expertise (63%).

In terms of service context, respondents indicated they needed at least a bit more:

- time to manage their caseload (71%)
- allocated time to assist consumers with non-clinical issues (70%)
- scope within their role to provide this support (64%)
- connections to their own service colleagues (60%).

The above findings identify features of legal capability as part of what this group of mental health workers feel they need more of. However, equally important were connection with other services and professionals and connection with community. While broad support for consumers is part of the remit of many respondents, many said they still needed at least a bit more time and scope within their roles to help consumers experiencing legal issues, as well as more time to manage their caseloads more generally.

**Figure 3:**
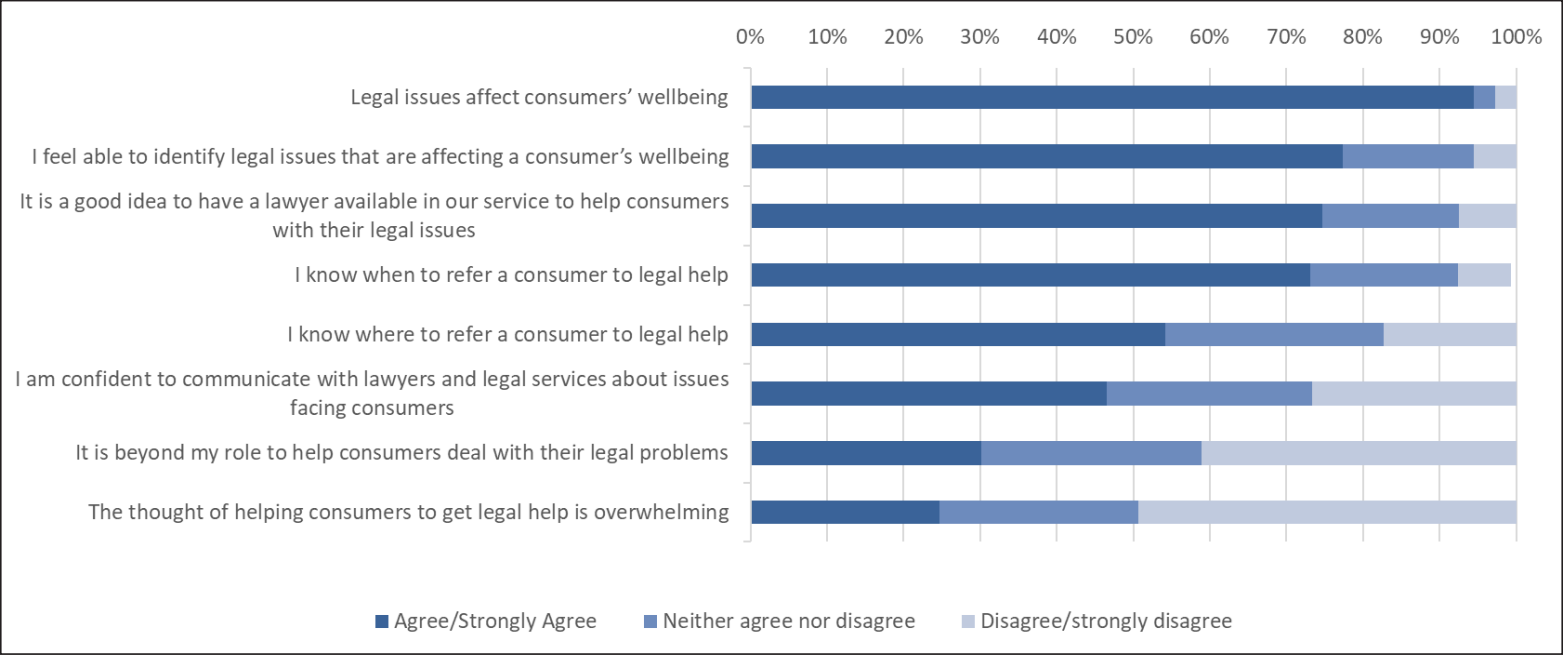
Perceptions of capability to support consumers with legal issues.

Respondents were also asked to respond to a series of statements about legal issues and their role in helping consumers with these issues. Their responses add context to the above observations (Figure 3).

First, nearly all (95%) agreed or strongly agreed with the statement that, ‘Legal issues affect consumers’ wellbeing’. Around three-quarters (77%) agreed they ‘feel able to identify legal issues that are affecting a consumer’s wellbeing’ and 73 per cent agreed they ‘know *when* to refer a consumer to legal help’. However, only 54% agreed that they ‘know *where* to refer a consumer to legal help’ and less than half (47%) agreed that they are ‘confident to communicate with lawyers and legal services about issues facing consumers’.

The statement, ‘It is beyond my role to help consumers deal with their legal problems’, received a mixed response, with 30% of respondents agreeing with the statement, another 29% neither agreeing nor disagreeing and 41 % disagreeing. There were no differences in this by respondent role type.

A quarter (25%) of respondents agreed that ‘The thought of helping consumers to get legal help is overwhelming’ while another quarter (26%) neither agreed nor disagreed. Half (49%) of all respondents disagreed with this statement.

## Discussion

Health justice partnerships integrate legal help into services that support health and wellbeing, to address the social and legal issues that contribute to poor health. HJPs provide accessible, timely legal help to individual clients in health and wellbeing settings who, evidence indicates, are more are more vulnerable to legal issues but less likely to seek help directly from legal services [2]. As observed in a recently released legal needs survey in Victoria:

Non-legal services play a critical role in supporting people facing justiciable [legal] problems. These include government or council bodies, organisations linked to work, professional and health services, dispute resolution bodies, and community organisations. People rely on them more frequently than legal services and they can be better positioned to support vulnerable populations. [5 p. 10]

Access to justice research also identifies the importance of ‘legal capability’ to recognise and respond to legal issues [3,10], most commonly in reference to those experiencing legal issues. But when intermediaries, such as mental health and wellbeing staff, are part of the pathway between those experiencing legal issue and remedies to those issues, they too require legal capability. Recognising this, health justice partnerships work to build the capability of and support for health practitioners to effectively identify legal issues affecting consumers, and to connect them with appropriate legal help [12,15].

This exploratory study of legal need in mental health support settings, and the self-assessed capability of mental health staff to respond to this need, was undertaken as a baseline before establishing health justice partnerships in a subset of a National provider’s mental health and wellbeing service sites. Survey respondents include support workers, peer support workers and clinicians from mental health services across Australia, all directly supporting people experiencing mental health issues.

### Legal need in mental health settings

The findings indicate that people are experiencing a wide range of socio-legal issues when they present to mental health services. The issues most commonly identified by staff were credit and debt issues, housing and tenancy issues, issues relating to family breakdown and family violence, and challenges interacting with government for support through social security and the National Disability Insurance Scheme. Of note, this set of legal issues reflect fundamental needs: income/financial security, care, housing and family, including family safety.

The findings are consistent with existing evidence about the common experience of legal problems among those with mental health issues and about the ‘clustering’ of legal issues together and with other life issues [2,8]. Recognising the bi-directional impact of mental health and legal issues [6,7,8], these findings indicate that staff are seeing consumers vulnerable to health harming social and legal issues: including issues that are generally beyond their primary expertise and remit to deal with. Existing literature identifies challenges faced by mental health practitioners in responding when their clients are experiencing social and legal issues interacting with their health [8,11]. HJP is a strategy to provide mental health services and staff with access to a broader range of tools to address these issues, building capability and connections through partnership with legal assistance services.

For legal assistance services these findings identify mental health services as places with concentration of people experiencing legal issues and who face barriers to accessing legal help and progressing matters without assistance. Such cohorts have been identified as a core priority for legal assistance services to reach and assist [16].

### Do workers have what they need to address legal issues that intersect with mental health?

This survey explored whether practitioners felt they had ‘enough’ knowledge, skills, trust, confidence, resources and connections to support consumers with legal issues, as well as organisational support such as remit, processes and resources.

Nearly 9 out of 10 respondents indicated that they *needed* more connections with and trust of legal services *and* connection to the communities they serve. This speaks to a bridging role for workers between consumers and the range of supports they may require when health and legal issues intersect [9,11]. More than three-quarters of all respondents also indicated they needed more processes, tools and resources to link consumers to other support services, together with time to spend on these issues and remit with their role. In terms of personal capability, most staff said they needed knowledge about the types of legal issues, skills and confidence to address legal issues.

Taken together, these responses indicate the central role of trusted intermediaries in building a pathway for consumers to legal help, through frontline health services. Underpinning trusted connections is the legal capability of staff (skills, knowledge and confidence) *together with* the commitment and practical support of their organisation.

### Experience with lawyers

Partnership needs to be built on trust and aligned interest. This can be particularly challenging when the partners coming together come from sectors as diverse as health and legal: sectors with separate evidence bases, different languages and potentially little insight into each other’s roles, ways of working and intended outcomes [3,17].

Among these mental health and wellbeing workers, one-third (32%) had never consulted lawyers to support their consumers while a further 28% said they had done so ‘a bit’. Forty per cent indicated that they had no referral relationships with legal services or that they did not know. Around half (49%) said they had informal referral relationships only, while 11% said they had formal referral relationships.

However, respondents also identified their interest in more connections with legal professionals and more knowledge about what they can provide. The experiences of health justice partnership indicate that these connections are a first step to building the understanding and aligned interests that provide a foundation for partnership.

### What do these results mean for legal and health services interested in partnership?

This study identifies mental health support services as sites accessed by people with co-occurring mental health and legal issues. Existing evidence points to how these legal and mental health issues can intersect [8]. This has implications for health and legal services and policy makers seeking to provide holistic, client-centred care for people experiencing complex intersecting need. It helps establish the need to build the capability and support for mental health services and staff to respond to this need when it arises, by drawing upon the expertise of other sectors who also seek to support this client group.

However, it only provides an overview. While this study indicates that the experience of legal issues is widespread and pervasive among service consumers, mental health services operate in specific sites and contexts. The specific legal issues arising in each setting will vary, as will the readiness and capability of services and staff to address those issues. The opportunities for connections with local legal services will also vary, together with their capacity to partner. Further, the appropriate response in any setting can sit along a spectrum of joint effort from networking and information exchange, through to relationships with more purposeful and shared investment such as partnership or integration [3,17]. This survey does not provide sufficient information to establish a response to legal need appropriate to specific service contexts.

To identify appropriate legal partners and build a partnership that responds to local needs and available infrastructure, the authors have conducted place-based assessments of legal need and opportunity, in the mental health service sites proposed for health justice partnership [18]. This methodology starts with the health service data to understand the profile of consumers served and connects this with evidence about the likely legal issues to arise [19]. This is complemented with insights from site staff about the legal needs arising in those sites, existing service connections, what staff may need to respond to those needs and their comfort with health justice partnership. This is used to facilitate discussions with potential legal partners.

This survey is also a valuable tool for the evaluation of health justice partnership, providing a benchmark against which service outcomes of health justice partnership can be assessed. Changes in health practitioner capability to address social issues affecting client health are a useful interim outcome of health justice partnership – a way to assess whether partnerships provide the tools and processes to support more integrated responses to complex need.

Finally, this study has focused on the self-assessed capability of mental health and wellbeing workers to identify and respond to legal issues. The capability and readiness of legal practitioners and services to partner in these spaces, is also critical, as is the funded capacity of the legal assistance sector to respond to legal need in these settings. Health justice partnership provides the opportunity to keep exploring how improved practitioner capability in each sector can contribute to the health and wellbeing of this shared client group.

## Conclusion

The findings of this exploratory study have implications for legal assistance services, for mental health and wellbeing services, their funders and policy makers. The study has identified the extent to which consumers in mental health settings are also experiencing legal issues – particularly around basic needs such as income/financial security, housing and family safety. These are issues which existing evidence indicates are likely to be affecting and affected by consumers’ health and wellbeing.

It also identified the capabilities and organisational support required by frontline staff in these mental health and wellbeing services to support clients experiencing these issues, recognising these issues affecting consumer health are beyond their primary expertise. If legal assistance services seek to rely on non-legal staff as ‘trusted intermediaries’ between themselves and clients, they also need to support the capability of these staff to do so. Health justice partnership provides the opportunity for health and legal services to complement each other’s work to provide client centred care for those experiencing intersecting health and legal needs.
